# Use of Genomics to Track Coronavirus Disease Outbreaks, New Zealand

**DOI:** 10.3201/eid2705.204579

**Published:** 2021-05

**Authors:** Jemma L. Geoghegan, Jordan Douglas, Xiaoyun Ren, Matthew Storey, James Hadfield, Olin K. Silander, Nikki E. Freed, Lauren Jelley, Sarah Jefferies, Jillian Sherwood, Shevaun Paine, Sue Huang, Andrew Sporle, Michael G. Baker, David R. Murdoch, Alexei J. Drummond, David Welch, Colin R. Simpson, Nigel French, Edward C. Holmes, Joep de Ligt

**Affiliations:** University of Otago, Dunedin, New Zealand (J.L. Geoghegan);; Institute of Environmental Science and Research, Wellington, New Zealand (J.L. Geoghegan, X. Ren, M. Storey, L. Jelley, S. Jefferies, J. Sherwood, S. Paine, S. Huang, J. de Ligt);; University of Auckland, Auckland, New Zealand. (J. Douglas, A. Sporle, A.J. Drummond, D. Welch);; Fred Hutchinson Cancer Research Centre, Seattle, Washington, USA (J. Hadfield);; Massey University, Auckland (O.K. Silander, N.E. Freed);; iNZight Analytics Ltd., Auckland (A. Sporle);; University of Otago, Wellington (M.G. Baker);; University of Otago, Christchurch, New Zealand (D.R. Murdoch);; Victoria University of Wellington, Wellington (C.R. Simpson);; University of Edinburgh, Edinburgh, Scotland, UK (C.R. Simpson);; Massey University, Palmerston North, New Zealand (N. French); T; he University of Sydney, Sydney, New South Wales, Australia (E.C. Holmes)

**Keywords:** 2019 novel coronavirus disease, coronavirus disease, COVID-19, severe acute respiratory syndrome coronavirus 2, SARS-CoV-2, viruses, respiratory infections, zoonoses, genomics, infectious disease, New Zealand

## Abstract

Real-time genomic sequencing has played a major role in tracking the global spread of severe acute respiratory syndrome coronavirus 2 (SARS-CoV-2), contributing greatly to disease mitigation strategies. In August 2020, after having eliminated the virus, New Zealand experienced a second outbreak. During that outbreak, New Zealand used genomic sequencing in a primary role, leading to a second elimination of the virus. We generated genomes from 78% of the laboratory-confirmed samples of SARS-CoV-2 from the second outbreak and compared them with the available global genomic data. Genomic sequencing rapidly identified that virus causing the second outbreak in New Zealand belonged to a single cluster, thus resulting from a single introduction. However, successful identification of the origin of this outbreak was impeded by substantial biases and gaps in global sequencing data. Access to a broader and more heterogenous sample of global genomic data would strengthen efforts to locate the source of any new outbreaks.

A genome of the novel severe acute respiratory syndrome coronavirus 2 (SARS-CoV-2) was published only 12 days after the virus was identified (*1*). This information was pivotal to the subsequent rapid development of diagnostic tests and identification of potential treatments (*2*,*3*). As of January 2021, ≈400,000 genomes of SARS-CoV-2 had been shared publicly (*4*). The underlying genome sequencing was performed so rapidly that during this infectious disease outbreak, virologic and epidemiologic data could be integrated in real time (*5*). Analysis of these data also played a role in informing the coronavirus disease (COVID-19) response by tracking the global spread and evolution of SARS-CoV-2, including identification of the number, source, and timing of introductions into individual countries, leading to a greater understanding of COVID-19 outbreaks around the world (*6*–*9*; A.D.S. Filipe et al., unpub. data, https://www.medrxiv.org/content/10.1101/2020.06.08.20124834v1; T. Seemann et al., unpub. data, https://www.medrxiv.org/content/10.1101/2020.05.12.20099929v1; L. Zhang et al., unpub. data, https://www.biorxiv.org/content/10.1101/2020.06.12.148726v1; J. Douglas et al., unpub. data, https://www.medrxiv.org/content/10.1101/2020.08.04.20168518v1).

As of January 2021, of the 219 countries that had reported positive cases of COVID-19 to the World Health Organization (WHO) (*10*), 65% (n = 142) had sequenced and shared SARS-CoV-2 genomes on the GISAID database (https://www.gisaid.org) (*4*). This immense global sequencing effort has enhanced ongoing genomic surveillance of the pandemic, including the monitoring of viral genetic changes of interest (L. Zhang et al., unpub. data, https://www.biorxiv.org/content/10.1101/2020.06.12.148726v1) and informing public health responses (*11*–*14*). Nevertheless, the number and proportion of SARS-CoV-2 genomes from COVID-19 case-patients that were sequenced, and genomes published, varies dramatically between countries and over time (Figure 1). For example, the COVID-19 Genomics UK Consortium (https://www.cogconsortium.uk) has led to the United Kingdom being the most represented sampling location, totaling ≈180,000 genomes and comprising 44% of the global dataset despite recording only ≈4% of the world’s positive cases (n = 3,669,658). Conversely, SARS-CoV-2 genomes sequenced in India represent just 1% of the global dataset but 11% of the world’s total reported cases (n = 10,677,710).

**Figure 1:**
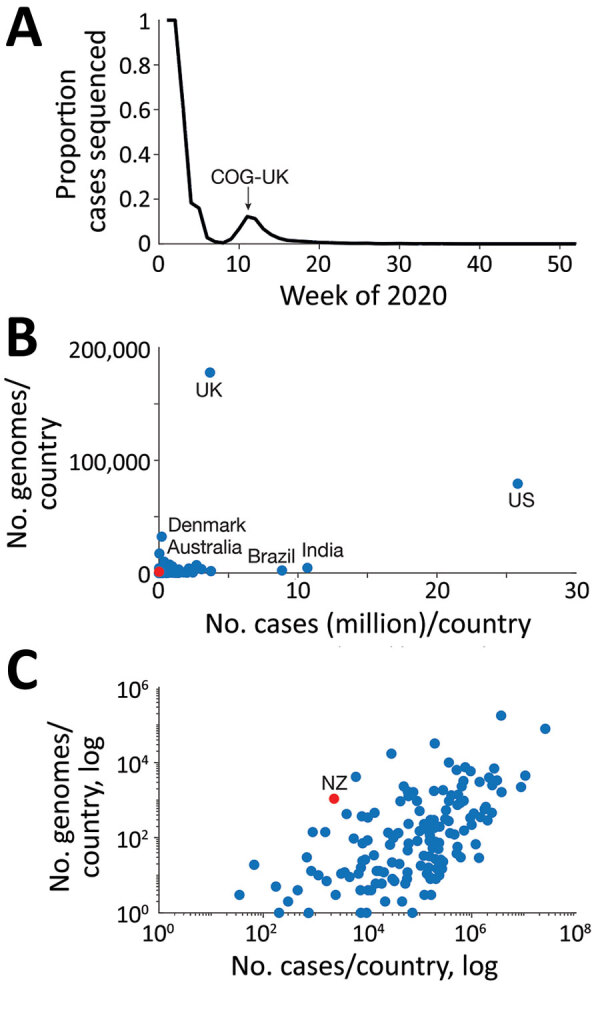
Sequenced and published genomes of global severe acute respiratory syndrome coronavirus 2 isolates. A) Proportion of global cases sequenced and shared on GISAID (https://www.gisaid.org) from December 2019 through January 2021, for which the second mode was largely driven by COG-UK as illustrated. B) Number of genomes sequenced and number of reported cases per country on a linear scale. Red, New Zealand (NZ); blue, other countries. C) Number of genomes sequenced and number of reported cases per country on a logarithmic scale. COG-UK, COVID-19 Genomics UK Consortium (https://www.cogconsortium.uk); UK, United Kingdom; US, United States.

Such disparate sequencing efforts can have major implications for data interpretation and must be carefully considered. Real-time sequencing of SARS-CoV-2 genomes has, however, been particularly useful for tracking the re-emergence of the virus in New Zealand. By June 2020, New Zealand had effectively eliminated COVID-19 in the community and positive cases were limited to those linked to managed quarantine facilities at the border (*7*,*15*; J. Douglas et al., unpub. data, https://www.medrxiv.org/content/10.1101/2020.08.04.20168518v1). After ≈100 days with no detected community transmission of COVID-19, on August 11, 2020, four new cases emerged with no apparent epidemiologic link to any known case. We used genomic sequencing of SARS-CoV-2 cases to investigate the probable origins of this outbreak, generating genomes for 78% (n = 140) of the 179 laboratory-confirmed samples from this outbreak.

We obtained nasopharyngeal samples positive for SARS-CoV-2 by real-time reverse transcription PCR (rRT-PCR) from public health medical diagnostics laboratories located throughout New Zealand. All samples had been de-identified before receipt. Under contract for the New Zealand Ministry of Health, the Institute of Environmental Science and Research (ESR) has approval to conduct genomic sequencing for surveillance of notifiable diseases.

## Methods

### Genomic Sequencing

Of 179 laboratory-confirmed samples of SARS-CoV-2 from the August 2020 outbreak in New Zealand, 172 were received by ESR for whole-genome sequencing. Genome sequencing of SARS-CoV-2 samples was performed as before (*7*). In brief, viral extracts were prepared from respiratory tract samples in which SARS-CoV-2 was detected by rRT-PCR by using World Health Organization–recommended primers and probes targeting the envelope and nucleocapsid genes. Extracted RNA from SARS-CoV-2–positive samples was subjected to whole-genome sequencing by following the ARTIC network protocol version 3 (https://www.protocols.io/view/ncov-2019-sequencing-protocol-v3-locost-bh42j8ye) and using the Massey University 1200-bp primer set (https://www.protocols.io/view/ncov-2019-sequencing-protocol-rapid-barcoding-1200-bh7hj9j6) (*16*).

We used 1 of the tiling amplicon designs to amplify viral cDNA prepared with SuperScript IV (ThermoFisher Scientific, https://www.thermofisher.com). Sequence libraries were then constructed by using Oxford Nanopore Ligation Sequencing and Native Barcoding Expansion kits for samples amplified with the ARTIC version 3 primer sets and the Oxford Nanopore Rapid Barcoding Kit for samples amplified with the 1,200-bp primer sets (https://nanoporetech.com). We used the 1,200-bp primers and rapid barcoding when genomes were required urgently. Libraries were sequenced by using R9.4.1 MinION flow cells (Oxford Nanopore). Near-complete (>90% recovered) viral genomes were subsequently assembled through reference mapping. Steps included in the pipeline are described in detail at https://github.com/ESR-NZ/NZ_SARS-CoV-2_genomics. The reads generated with Nanopore sequencing using ARTIC primer sets (version 3) were mapped and assembled by using the ARTIC bioinformatics Medaka pipeline version 1.1.0. In total, 140 of 172 genomes from the August 2020 outbreak passed quality control. All data are available on GISAID (https://www.gisaid.org).

### Phylogenetic Analysis

All SARS-CoV-2 genomes from humans, assigned to the B.1.1.1. lineage in the pangolin nomenclature (*17*), were obtained from GISAID (*4*) (n = 7,363 as of January 26, 2021) and subsampled to include 1,996 most recent-in-time sequences to the August 2020 New Zealand outbreak along with 4 outgroup (non-B.1.1.1.) sequences. Sequences were aligned with those from the August 2020 outbreak (n = 140) by using MAFFT version 7 (*18*) and the FFT-NS-2 progressive alignment algorithm (Appendix 1). Bayesian phylogenetic analyses were performed by using BEAST 2.5 (*19*). We used a strict clock model with an HKY (Hasegawa, Kishino, and Yano) substitution model (estimated frequencies) for each codon position and 1 for noncoding positions. We used the Bayesian skyline model (*20*) as a tree to allow effective population sizes to change over time intervals. These components of the model and their prior distributions have been previously used (J. Douglas et al., unpub. data, https://www.medrxiv.org/content/10.1101/2020.08.04.20168518v1). Phylogenetic trees were annotated by using FigTree version 1.4 (http://tree.bio.ed.ac.uk/software/figtree) and Tree of Life version 4 (*21*).

## Results

Of the virus genomes generated in real time for 78% of cases in this cluster, from August 11 through September 14, 2020, when the last case in this outbreak was reported, the maximum distance among the genome was 5 single-nucleotide polymorphisms. When we compared the genomes from patients in the August 2020 New Zealand outbreak with sequenced genomes from patients affected by the first COVID-19 wave in New Zealand and those in quarantine facilities, we found no link. Most available sequence data from case-patients in New Zealand quarantine facilities indicated virus lineages different from those of the August 2020 outbreak. However, this observation was of limited value given that only 42% of case-patients in those quarantine facilities had adequate viral RNA for successful genomic sequencing. To determine the likely origins of this outbreak, we compared genomes from the new community outbreak to the global dataset.

**Figure 2:**
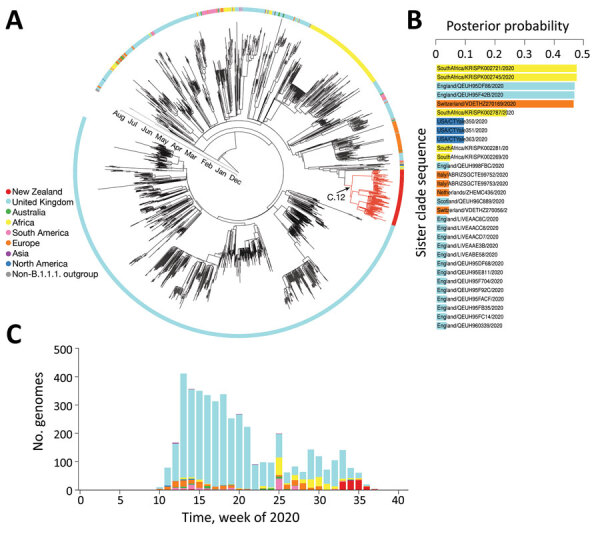
Genomic sequence analyses of global severe acute respiratory syndrome coronavirus 2 isolates. A) Maximum-clade credibility phylogenetic tree of 2,000 subsampled global genomes (1,996 most recently sampled B.1.1.1. plus 4 non-B.1.1.1. used as an outgroup) with an outer ring colored by sampling region. B) Posterior probability of genomes within the sister clade to that of the August 2020 outbreak in New Zealand, color coded by sampling location. C) Proportion of genomes within lineage B.1.1.1. in the global dataset over time, color-coded by sampling location.

An initial genomic sequence analysis found that the reemergence of COVID-19 in New Zealand was caused by a SARS-CoV-2 from the (now ancestral) lineage B.1.1.1 of the pangolin nomenclature (*17*). Of the countries that have contributed SARS-CoV-2 data, 30% had genomes of this lineage. Remarkably, 80% of B.1.1.1. genomes were from the United Kingdom and were generated during March 2020–January 2021; however, most samples were collected during the first wave of disease in the United Kingdom (Figure 2). Phylogenetic analysis of the most recently sampled B.1.1.1. genomes identified genomes from South Africa, England, and Switzerland in August as the most likely to be contained within the sister clade (Figure 2); these genomes were the closest sampled genomic relatives of the viruses associated with the August 2020 outbreak in New Zealand (Appendix 2). Because of the dynamic nature of the pangolin lineage nomenclature, genomes sampled from the August 2020 outbreak in New Zealand are now distinctly classified as lineage C.12, which is now extinct.

Additional Bayesian analysis estimated that the outbreak originated 10 days before the first transmission event; the 95% highest posterior density was 0–25 days. We also estimated that the first transmission event in the outbreak occurred during July 22–August 13, 2020 (95% highest posterior density mean date of August 2). Epidemiologic data showed that 2 confirmed case-patients linked to the outbreak had a symptom onset date of July 31, although the most probable sampled genomes within the sister clade were sampled later, August 6–28. Hence, it is unlikely that the currently available global genomic dataset contains the source of this outbreak.

## Discussion

Genomic epidemiologic analysis of the possible origins of the COVID-19 re-emergence in New Zealand in August 2020 was inconclusive, probably because of missing genomic data within the quarantine border facilities and in the global dataset. A glimpse into the genomic diversity probably omitted from the global dataset can be seen in the genomes sequenced in New Zealand from SARS-CoV-2–positive quarantined case-patients, comprising citizens and residents returning from across the globe. For example, 12 SARS-CoV-2 genomes from persons returning to New Zealand from India who arrived on the same flight fell across at least 4 genomic lineages and comprised sequence divergence of up to 34 single-nucleotide polymorphisms (https://www.nextstrain.org). This divergence represented far more genomic mutations than was observed in New Zealand during the first outbreak in March–May 2020 (*7*). Such a high level of diversity in just a small sample of SARS-CoV-2–positive case-patients from India suggests that the currently available genomic data fail to encompass the true diversity that existed locally, let alone globally.

The genome sequences identified after the re-emergence of SARS-CoV-2 in New Zealand in August 2020 exemplified one of the most complete genomic datasets for a specific outbreak compiled to date, comprising 78% of positive case-patients (140 of 179 total case-patients SARS-CoV-2 positive by PCR). Real-time genomic sequencing quickly informed track-and-trace efforts to control the outbreak, setting New Zealand on track to eliminate the virus from the community for the second time. The rapid genome sequencing of positive samples provided confidence to public health teams regarding links to the outbreak and identified that cases and subclusters were linked to a single genomic lineage, resulting from a single introduction event. Indeed, the timing and length of lockdown measures were partly informed on the basis of these data. Overall, real-time viral genomics has played a pivotal role in eliminating COVID-19 from New Zealand and has since helped prevent additional regional lockdowns, leading to substantial economic savings.

Nevertheless, the biased nature of global sampling, including the contribution of very few genome sequences from certain geographic locations, clearly limited the power of genomics to attribute the geographic origin of the August 2020 outbreak in New Zealand. We therefore advocate that potential sampling biases and gaps in available genomic data be carefully considered whenever attempting to determine the geographic origins of a specific SARS-CoV-2 outbreak. Analyses should consider all available evidence, including that from genomic and epidemiologic sources.

Appendix 1 List of genomes obtained from GISAID used in analysis of severe acute respiratory syndrome coronavirus 2.

Appendix 2 Phylogenetic tree of severe acute respiratory syndrome coronavirus 2 genomes.

## References

[1] Holmes EC. Novel 2019 coronavirus genome [cited 2021 Jan 20]. https://virological.org/t/novel-2019-coronavirus-genome/319

[2] Shin MD, Shukla S, Chung YH, Beiss V, Chan SK, Ortega-Rivera OA, et al. COVID-19 vaccine development and a potential nanomaterial path forward. Nat Nanotechnol. 2020;15:646–55. 10.1038/s41565-020-0737-y 32669664

[3] Stebbing J, Phelan A, Griffin I, Tucker C, Oechsle O, Smith D, et al. COVID-19: combining antiviral and anti-inflammatory treatments. Lancet Infect Dis. 2020;20:400–2. 10.1016/S1473-3099(20)30132-8 32113509 PMC7158903

[4] Elbe S, Buckland-Merrett G. Data, disease and diplomacy: GISAID’s innovative contribution to global health. Glob Chall. 2017;1:33–46. 10.1002/gch2.1018 31565258 PMC6607375

[5] Hadfield J, Megill C, Bell SM, Huddleston J, Potter B, Callender C, et al. Nextstrain: real-time tracking of pathogen evolution. Bioinformatics. 2018;34:4121–3. 10.1093/bioinformatics/bty407 29790939 PMC6247931

[6] Bedford T, Greninger AL, Roychoudhury P, Starita LM, Famulare M, Huang M-L, et al.; Seattle Flu Study Investigators. Cryptic transmission of SARS-CoV-2 in Washington state. Science. 2020;370:571–5. 10.1126/science.abc0523 32913002 PMC7810035

[7] Geoghegan JL, Ren X, Storey M, Hadfield J, Jelley L, Jefferies S, et al. Genomic epidemiology reveals transmission patterns and dynamics of SARS-CoV-2 in Aotearoa New Zealand. Nat Commun. 2020;11:6351. 10.1038/s41467-020-20235-8 33311501 PMC7733492

[8] Candido DS, Claro IM, de Jesus JG, Souza WM, Moreira FRR, Dellicour S, et al.; Brazil-UK Centre for Arbovirus Discovery, Diagnosis, Genomics and Epidemiology (CADDE) Genomic Network. Evolution and epidemic spread of SARS-CoV-2 in Brazil. Science. 2020;369:1255–60. 10.1126/science.abd2161 32703910 PMC7402630

[9] Eden J-S, Rockett R, Carter I, Rahman H, de Ligt J, Hadfield J, et al. 2019-nCoV Study Group. An emergent clade of SARS-CoV-2 linked to returned travellers from Iran. Virus Evol. 2020;6:a027. 10.1093/ve/veaa027 PMC7147362 32296544

[10] Dong E, Du H, Gardner L. An interactive web-based dashboard to track COVID-19 in real time. Lancet Infect Dis. 2020;20:533–4. 10.1016/S1473-3099(20)30120-1 32087114 PMC7159018

[11] Oude Munnink BB, Nieuwenhuijse DF, Stein M, O’Toole Á, Haverkate M, Mollers M, et al.; Dutch-Covid-19 response team. Rapid SARS-CoV-2 whole-genome sequencing and analysis for informed public health decision-making in the Netherlands. Nat Med. 2020;26:1405–10. 10.1038/s41591-020-0997-y 32678356

[12] Kalinich CC, Jensen CG, Neugebauer P, Petrone ME, Peña-Hernández M, Ott IM, et al. Real-time public health communication of local SARS-CoV-2 genomic epidemiology. PLoS Biol. 2020;18:e3000869. 10.1371/journal.pbio.3000869 32822393 PMC7467297

[13] Rockett RJ, Arnott A, Lam C, Sadsad R, Timms V, Gray K-A, et al. Revealing COVID-19 transmission in Australia by SARS-CoV-2 genome sequencing and agent-based modeling. Nat Med. 2020;26:1398–404. 10.1038/s41591-020-1000-7 32647358

[14] Bauer DC, Tay AP, Wilson LOW, Reti D, Hosking C, McAuley AJ, et al. Supporting pandemic response using genomics and bioinformatics: A case study on the emergent SARS-CoV-2 outbreak. Transbound Emerg Dis. 2020;67:1453–62. 10.1111/tbed.13588 32306500 PMC7264654

[15] Jefferies S, French N, Gilkison C, Graham G, Hope V, Marshall J, et al. COVID-19 in New Zealand and the impact of the national response: a descriptive epidemiological study. Lancet Public Health. 2020;5:e612–23. 10.1016/S2468-2667(20)30225-5 33065023 PMC7553903

[16] Freed NE, Vlková M, Faisal MB, Silander OK. Rapid and inexpensive whole-genome sequencing of SARS-CoV-2 using 1200 bp tiled amplicons and Oxford Nanopore Rapid Barcoding. Biol Methods Protoc. 2020;5:a014. 10.1093/biomethods/bpaa014 33029559 PMC7454405

[17] Rambaut A, Holmes EC, O’Toole Á, Hill V, McCrone JT, Ruis C, et al. A dynamic nomenclature proposal for SARS-CoV-2 lineages to assist genomic epidemiology. Nat Microbiol. 2020;5:1403–7. 10.1038/s41564-020-0770-5 32669681 PMC7610519

[18] Katoh K, Standley DM. MAFFT multiple sequence alignment software version 7: improvements in performance and usability. Mol Biol Evol. 2013;30:772–80. 10.1093/molbev/mst010 23329690 PMC3603318

[19] Bouckaert R, Vaughan TG, Barido-Sottani J, Duchêne S, Fourment M, Gavryushkina A, et al. BEAST 2.5: An advanced software platform for Bayesian evolutionary analysis. PLOS Comput Biol. 2019;15:e1006650. 10.1371/journal.pcbi.1006650 30958812 PMC6472827

[20] Drummond AJ, Rambaut A, Shapiro B, Pybus OG. Bayesian coalescent inference of past population dynamics from molecular sequences. Mol Biol Evol. 2005;22:1185–92. 10.1093/molbev/msi103 15703244

[21] Letunic I, Bork P. Interactive Tree Of Life (iTOL) v4: recent updates and new developments. Nucleic Acids Res. 2019;47(W1):W256–9. 10.1093/nar/gkz239 30931475 PMC6602468

