# Supplementary material for: Use of Genomics to Track Coronavirus Disease Outbreaks, New Zealand
Source: Emerg Infect Dis. 2021 May;27(5):1317–22. doi: 10.3201/eid2705.204579 (PMC8084492; doi:10.3201/eid2705.204579)
Supplement: Appendix 2 — Phylogenetic tree of severe acute respiratory syndrome coronavirus 2 genomes. [file 20-4579-Techapp-s2.pdf]

Article DOI: https://doi.org/10.3201/eid2705.204579

Use of Genomics to Track Coronavirus Disease Outbreaks, New Zealand

Appendix

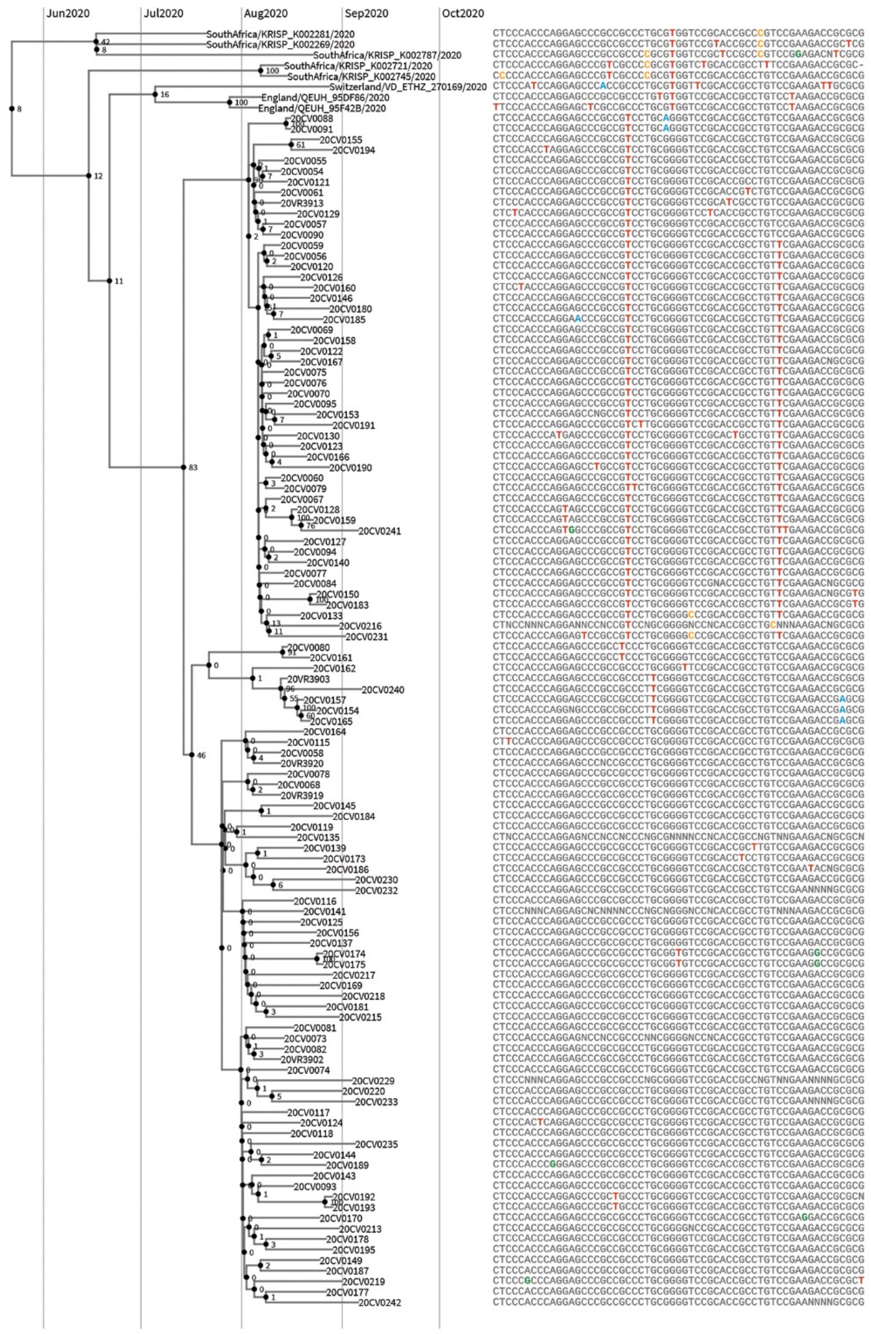

Appendix Figure. Phylogenetic tree as presented in Figure 2 of the main article, although only taxa from the recent New Zealand outbreak are included, alongside five closest outgroup taxa. Clade posterior support values (percentages) are labelled on internal nodes. Alignment positions are only included in the figure if the site contains at least two variants among the taxa considered (ambiguous nucleotides are ignored from the count). The minority character in each column is colored.
